# Supplementary material for: Differential Disrupting Effects of Prolonged Low-Dose Exposure to Dichlorodiphenyltrichloroethane on Androgen and Estrogen Production in Males
Source: Int J Mol Sci. 2021 Mar 19;22(6):3155. doi: 10.3390/ijms22063155 (PMC8003643; doi:10.3390/ijms22063155)
Supplement: Supplementary file 1 [file ijms-22-03155-s001.pdf]

Supplement

Table S1. Physical and chemical characteristics of o,p-DDT.

Property	Information
Synonyms	2,4'-DDT<br>1,1,1-trichloro-2-(*o*-chlorophenyl)-2-(*p*-chlorophenyl)ethane<br>*o,p'*-dichlorodiphenyl-trichloroethane
Chemical formula	$C_{14}H_9Cl_5$
Chemical structure	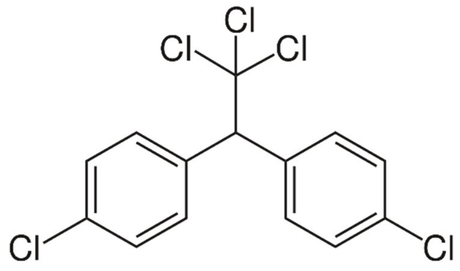
CAS registry number	789-02-6
Molecular weight	354.49
Physical state	Solid, crystalline powder
Color	white
Odor	odorless
Density	0.985 g/cm3 at 20°C
Water solubility	85 µg/l at 25°C
